# Tale of a multifaceted co-activator, hADA3: from embryogenesis to cancer and beyond

**DOI:** 10.1098/rsob.160153

**Published:** 2016-09-07

**Authors:** Vaibhav Chand, Deeptashree Nandi, Anita Garg Mangla, Puneet Sharma, Alo Nag

**Affiliations:** Department of Biochemistry, University of Delhi South Campus, New Delhi, 110021, India

**Keywords:** ADA3, co-activator, genomic stability, development, cancer

## Abstract

Human ADA3, the evolutionarily conserved transcriptional co-activator, remains the unified part of multiple cellular functions, including regulation of nuclear receptor functions, cell proliferation, apoptosis, senescence, chromatin remodelling, genomic stability and chromosomal maintenance. The past decade has witnessed exciting findings leading to considerable expansion in research related to the biology and regulation of hADA3. Embryonic lethality in homozygous knockout Ada3 mouse signifies the importance of this gene product during early embryonic development. Moreover, the fact that it is a novel target of Human Papillomavirus E6 oncoprotein, one of the most prevalent causal agents behind cervical cancer, helps highlight some of the crucial aspects of HPV-mediated oncogenesis. These findings imply the central involvement of hADA3 in regulation of various cellular functional losses accountable for the genesis of malignancy and viral infections. Recent reports also provide evidence for post-translational modifications of hADA3 leading to its instability and contributing to the malignant phenotype of cervical cancer cells. Furthermore, its association with poor prognosis of breast cancer suggests intimate association in the pathogenesis of the disease. Here, we present the first review on hADA3 with a comprehensive outlook on the molecular and functional roles of hADA3 to provoke further interest for more elegant and intensive studies exploring assorted aspects of this protein.

## 1. Introduction

A breakthrough in understanding transcriptional control mechanisms was furnished by the discovery of co-activators, a diverse array of cellular factors that connect sequence-specific DNA-binding activators to the general transcriptional machinery, or that help activators and the transcriptional apparatus to navigate through the constraints of chromatin. Among several co-activators known so far, human ADA3 is an ambidextrous protein. It exhibits tremendous variability in its interacting complexes to regulate a number of cellular processes. Since its discovery, new findings have constantly stimulated researchers to put together the puzzle pieces, revealing the biology of hADA3. This review examines the evidence that established ADA3 as a *bona fide* component of co-activator complex across the species, outlining recent developments in the field to provide unprecedented insight into the ever-expanding functional characteristics of hADA3, and discusses important directions for future research.

## 2. Discovery of ADA3

**Figure 1.**
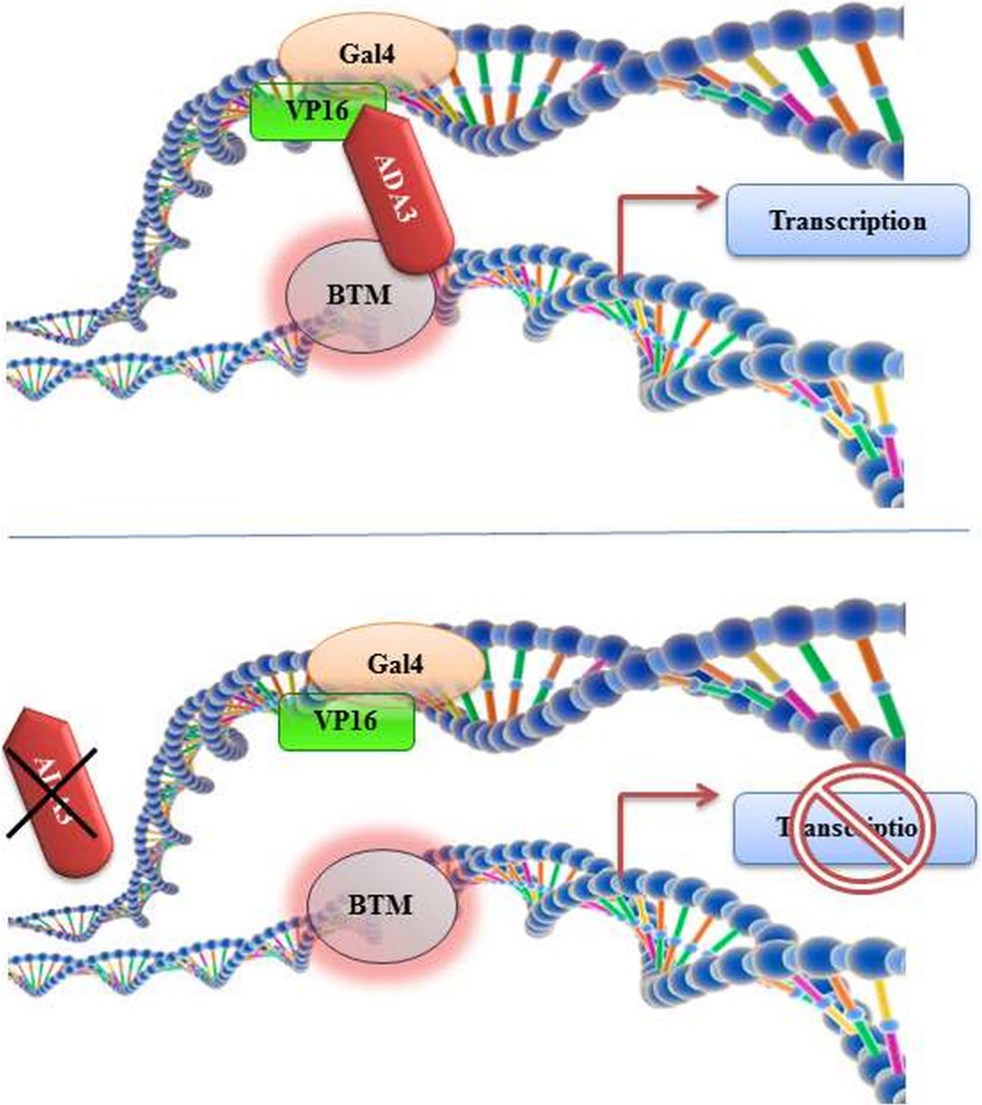
Discovery of ADA3 and its consequential role in transcriptional activation. ADA3 was isolated as a transcriptional co-activator using a Gal4-VP16 system, where removal of ADA3 disrupted transcriptional activation by abrogating the ADA3-mediated bridging interaction between the general factors and the distal activators.

The discovery and designation of yADA3 as co-activator was originally reported by Berger *et al.* in 1992 [1]. Slow-growing and temperature-sensitive (fails to grow at 37°C) yeast mutants were observed to avert the cytotoxic effect of GAL4-VP16 constitutive expression by dismantling the general transcription machinery (figure 1) [2].

Mutational studies and yeast genomic library search led to the discovery of yAda3 gene, which encodes for a 702-amino-acid-containing protein identified to restore the toxic property of GAL4-VP16. Studies of yADA3 established its pivotal role in chromatin remodelling by recruiting histone acetyl transferase (HAT) complexes to the nucleosomes. yADA3 also remains the indivisible component of yeast ADA and SAGA-HAT complexes. Intriguingly, *Drosophila* ADA3 was discovered during the characterization of dGcn5-associated HAT complex. dADA3 was found to be a central member of Gcn5-containing *N*-acetyltransferase HAT complex, and it controls the embryonic development of *D. melanogaster*. Further characterization intimates that dADA3 is part of dSAGA like HAT complexes and plays an indispensable role in the maintenance of chromatin dynamicity. Study of one such mammalian multi-protein HAT complex containing PCAF led to the identification of hADA3 as novel interacting partner [3]. Similar to yeast ADA and SAGA complex, hADA3 also plays a crucial role in enhancing the HAT activity of PCAF complex in mammals [3].

## 3. Biological functions of ADA3

Human ADA3 is a multifaceted protein beyond being merely a transcriptional co-activator. Accumulating evidence suggests that, apart from its co-activator function, ADA3 participates in countless other biological soirées such as chromatin remodelling, cellular proliferation, cellular senescence, DNA damage response (DDR) and, more importantly, it plays a decisive role in embryonic development. In the forthcoming section, we present a few momentous testimonies supporting the role of ADA3 in a myriad of processes unequivocally important for normal functioning of the body.

### 3.1. Role of ADA3 in histone acetylation and chromatin remodelling

The presence of ADA3 in ADA, SAGA, STAGA, TFTC and PCAF like HAT complexes across species highlights its role in the acetylation of histones and regulation of chromatin dynamicity. Additionally, ADA3 deletion study suggests that ADA3 possesses two functionally non-overlapping domains of which the amino terminal domain is required for binding with protein complexes present at upstream activation sequence (UAS), whereas the carboxyl terminal domain is required to recruit ternary complex of ADA2 and Gcn5 (HATs)—this was confirmed by co-IP study [4]. Yeast ADA2, ADA3 and GCN5 form a catalytic core of the ADA and SPT-ADA-GCN5-acetyltransferase HAT complex, which is necessary and sufficient for *in vitro* nucleosomal HAT activity and lysine specificity of the intact HAT complexes. This suggests the possibility of ADA3 participation in recognition of histone tails in a nucleosomal context, which warrants further investigation. ADA3 has been shown to regulate the substrate specificity of GCN5 HAT and thus enable GCN5 to act on its physiological substrate, chromatin [5]. The distinct times of arrest observed with Gcn5-null and Ada3-null embryos point towards the possibility of ADA3-mediated roles in early development through complexes in which GCN5 is not a critical component or is functionally redundant with other HATs [6].

In *Drosophila ADA3* mutants, acetylation at histone H3 K9, K12 and K14, but not K18, is significantly reduced. Moreover, reduction of acetylation-dependent phosphorylation of H3 S10 in dADA3 mutants illustrates the role of dADA3 within HAT complexes in acetylation of H3 and H4 at specific residues [7]. A recent study also identifies PHF21A as an ADA3 interacting partner, a crucial component of BRAF35/histone acetyl deacetylase (HDAC) complex and a recognized chromatin remodeller. PHF21A introduces specific modifications in histones and also inhibits de-methylation of Lys4 in Histone-3. This interaction further accentuates the role of ADA3 in chromatin remodelling and modulating histone modifications [8]. ADA3 has also been identified in a number of alternate complexes, such as STAGA complex in mammals, which includes both hGCN5 and PCAF [3,9,10]. In coordination with this evidence, Ada3 knockout MEFs displayed drop in core histone acetylation at H2A-K5, H2B-K5, H3-K9, H3-K56 and H4-K8. Ada3^−/−^ MEFs also show downregulation of p300, a known interacting partner of hADA3, and PCAF expression, in contrast to control MEFs, and this provides a logical explanation about the reduction in global histone acetylation [6]. Studies have shown ADA3 binds to and stabilizes the tumour suppressor protein p53 and is essential for p300-mediated p53 acetylation. ADA3 is required for HAT recruitment to oestrogen receptors and their transcriptional activation function. Loss of mammalian ADA3 led to a dramatic decrease in acetylation of the core histones that play a role in progression of cell cycle, gene expression patterns and other essential physiological functions of the cell.

Reversible phosphorylation is also an important step of chromatin remodelling and regulation of transcription. By employing yeast two hybrid system, PPP2R5D and PPP1R7 were acknowledged as novel interacting partners of hADA3 [8]. PPP2R5D and PPP1R7 are the components of NuA4 HAT complex and control the chromatin remodelling through dephosphorylating activity. Furthermore, these are the components of PP2A and PP1 family phosphatases, which suggests a new role of hADA3 in regulation of chromatin remodelling by regulating the extent and patterns of phosphorylation.

### 3.2. ADA3 as a co-activator

**Figure 2.**
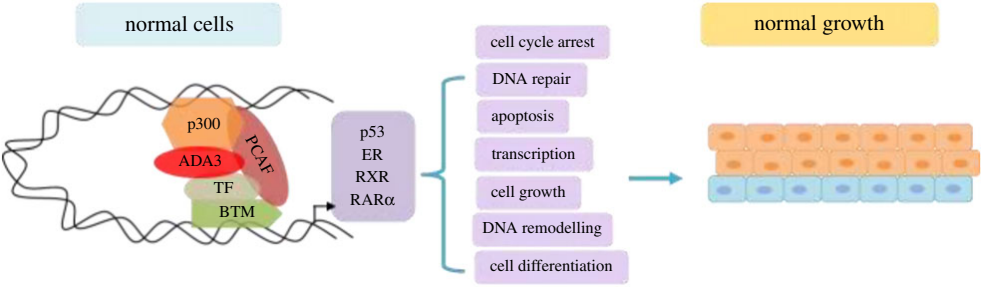
Function of hADA3 as a co-activator. hADA3 is part of the BTM and acts as a transcriptional co-activator to conduit the UAS to the BTM for the transcriptional activation of p53, ER, RAR, RXR, etc., in order to regulate various cellular functions that are crucial for normal cellular growth.

Transcriptional co-activators can bind to target transcription factors (TF) in either ligand-dependent or -independent manner. Although many of them are capable of directly interacting with the basal transcriptional machinery (BTM), a number of transcriptional co-activators exhibit enzymatic function intrinsically linked to gene regulation, such as the nucleosome-modifying HAT or HDAC activities [11,12]. Although ADA3 appears to satisfy these aforesaid criteria, its functional significance as a co-activator was further substantiated by studies where yADA3 was found to act as transcriptional co-activator of p53, oestrogen receptor-α (ERα) and Retinoic X Receptor-α (RXRα) (figure 2) [13]. HPV16 E6, by targeting hADA3 for degradation, abrogates the RXRα-mediated transactivation, implicating disruption of hADA3-dependent retinoid receptor function in E6 oncogenesis. Considering the evolutionary conservation of hADA3, it is likely that viral oncoprotein-mediated inactivation of its function plays an important role in oncogenesis [14]. ADA3 seems to bind to RAR subtypes with different affinity. In particular, RARγ shows better interaction with ADA3 when compared with RARα, possibly owing to the ω loop in the RARγ, which is absent in RARα and RARβ. This loop might facilitate the conformational change of the RAR/ADA3 interaction [15].

Benecke *et al.* [16] reported the first interaction of mouse ADA3 containing TBP-free TAF-containing (TFTC) complexes with nuclear receptor ERα, whereas human ADA3 was later shown by Meng *et al*. [17] to interact with ERα and ERβ in ligand-dependent manner. Oestrogen-dependent transcriptional regulation of ER downstream targets by hADA3 classifies it to be a member of steroid receptor co-activator (SRC) family. Additionally, ADA3 was found to control transcriptional activation of T3 hormone response element by hTRb1 (human T3 receptor b1) in ligand-dependent manner through association with GCN5 and ADA2 adaptor proteins, suggesting that ADA3 is an integral part of the HAT complexes and plays a central role in the transcriptional regulation as a co-activator [18]. Thus, based on these findings, it can be speculated that high-risk viral oncoproteins like HPV16E6 can easily hijack this multifunctional co-activator to deregulate transcriptional machinery leading to abnormal proliferation of cells (figure 6) [17,19]. Recent findings show enhanced c-Myc expression mediated by STAGA. c-Myc regulates transcription of Skp2, a component of SCF (Skp2 cell–cycle-associated E3 ligase) that regulates p27 levels. ADA3 being a component of STAGA, cell cycle-associated Myc transcription is ADA3-dependent, and thus ADA3 is required for Skp2 transcription and p27 stability. STAGA has also been shown to associate with c-Myc on c-Myc target gene promoters and is required for efficient c-Myc-mediated transcriptional activation [6]. Whereas some co-activators, such as p300 and CBP, have intrinsic HAT activity, and thus can directly participate in chromatin remodelling, other proteins function as co-activators by recruiting crucial components of the BTM as part of a larger complex. Since ADA3 lacks a HAT domain, it is worthwhile to determine which protein interacts with ADA3 to constitute a functional co-activator. Observations such as the presence of hGCN5, as well as an hGCN5-related gene product P/CAF in hADA3-containing complexes, direct interaction of P/CAF with p300/CBP and interaction of hADA3 itself with p300, suggest that hADA3 may form multiple, distinct co-activator complexes, similar to yADA3. Thus, it is likely that additional transcriptional activators will emerge as targets of ADA3 co-activator function [13].

Though the functional role of ADA3 as a transcriptional co-activator is well established, evidence demonstrating interaction of hADA3 with PHD Finger Protein 21A (PHF21A), a component of BHC-HDACs, which plays a crucial role in suppression of neuronal genes in non-neuronal cells, pointed towards a co-repressor function of hADA3 [20]. Moreover, hADA3 was found to subdue the *in vitro* transcription of MDM2 by newly identified interacting partners apoptosis antagonizing transcription factor (AATF), PPP2R5D and PPP1R7, including PHF21A, fortifying the transcriptional co-repressor activity of hADA3 [8]. However, this alternative and conflicting functional role of ADA3 needs to be documented further.

### 3.3. ADA3 in cell proliferation and mitosis

In 1992, with the identification of ADA3, experimental evidence in that initial report implied a role of ADA3 in induction of cellular growth and proliferation [1]. Though in the preliminary report yADA3 mutants displayed slow growth rate when compared with the wild-type strains, the first confirmative evidence came from the study in which hAda3 knockdown ER-positive MCF7 cells showed reduced cellular proliferation [10]. This study demonstrated a significant role of ADA3 in recruitment of HAT to oestrogen-responsive gene promoters and induction of ER-dependent proliferation of breast cancer cells [10]. This was in accordance with earlier findings showing direct interaction of ADA3 with ER and enhancement of ER-responsive genes [17]. Wu *et al*. [21] reported a key role of yADA3 in regulation of cellular proliferation and transient transcriptional induction following stress elimination in systems confronted with nutrient stress. In their experiments, employing a *Saccharomyces cerevisiae* model, they demonstrated that ADA3 is essential in transcriptional induction of CLN3, a G1 cyclin-encoding gene, which plays a pivotal role in rapid cellular growth, and progression through the cell cycle, whereas loss of ADA3 impedes the process [21]. Additionally, experimental evidences of Ada3 KO MEFs that display a delayed progression of cells from G1 to S phase and G2/M to G1 phase further supported prior results. It was found to be a consequence of p27 accumulation, a target of Myc oncoprotein. [6]. Another report highlighted a link between hADA3 and β-catenin in regulation of cellular proliferation [22]. Given the irreparable role of β-catenin in development, hAda3 KO embryonic lethality in mouse is somewhat rationalized, although it still remains a point to unravel. Studies have also revealed evidence for requirement of ADA2 and hADA3 in ATAC complex which plays a pivotal role in control of mitosis [23]. Since ATAC complex helps in acetylation of cyclin A/Cdk2 in early M-phase, this establishes a link between hADA3 and normal cell cycle progression [23]. Additionally, ADA3 is also required to control the expression of T3 response element genes, which are known to control cell growth, development and homeostasis [18].

The centromere proteins include a complex set-up of highly coordinated proteins that are involved in faithful segregation of chromosomes. ADA3 reportedly associates with CENP-B, and this interaction persists throughout all phases of the cell cycle and is a climacteric event in controlled cellular proliferation. It is known that cells with endogenous ADA3 ablation reveal a cell proliferation defect that is rescued by overexpression of ADA3. A substantially important observation was that treating these cells with an ADA3 mutant, with the CENP-B interacting region removed, could not restore the normal phenotype. Ada3 knockdown also significantly reduced centromere binding of CENP-B. Analyses of anaphase chromosomes in Ada3-deleted cells demonstrated heightened chromosome mis-segregation events compared with control cells, suggesting an essential role of ADA3 in genomic stability [24]. Thus, this study attested to the novel role of ADA3 in regulation of mitosis through recruitment of CENP-B to the centromere and underscored the role of this interaction in abnormal cell proliferation causing oncogenesis.

### 3.4. ADA3 in cellular senescence

Cellular senescence or Hayflick's phenomenon is a state of irreversible growth arrest following stress [25]. Owing to the complexity of the process and technical limitations, comprehension of mechanisms of cellular senescence still remains elusive. Studies using hTERT immortalized mammary epithelial cells (hTERT-MEC) have shown that overexpression of p14ARF induces p53-dependent senescence [26]. The result also indicated the direct correlation between the extent of senescence and level of p53 acetylation. hADA3, being a component of HAT complex, modulates acetylation of p53, leading to activation of p14ARF signalling and subsequent induction of cellular senescence [26,27]. This was further confirmed when depletion of hADA3 abrogated the p14ARF-induced senescence in hTERT-MEC cells. Truncated hADA3 protects immortal hTERT-MEC from p14ARF-induced senescence. hADA3-specific siRNA inhibits p300-mediated acetylation of p53. The participation of hADA3 in p53-dependent senescence was further substantiated by employing E6 mutant that can degrade hADA3 while p53 remains untouched [27]. Hence, in addition to its ability to induce p53 ubiquitination, hADA3 degradation represents another mechanism for HPV16 E6-mediated p53 inhibition.

Both truncated hADA3 and the HPV16 E6 mutant, which degrades hADA3 but not p53, attenuated p14ARF-induced senescence in cells that maintain expression of p14ARF and p53. This is achieved by inhibition of p53 acetylation and inhibition of accumulation of p21cip1. Likewise, previous reports have shown overexpression of p21cip1 induces senescence in human fibroblasts, whereas p21cip1 null human fibroblasts are resistant to p14ARF-mediated senescence. Evidence also indicated that hAda3 knockdown reduced the level of p53 acetylation by p300 at the K382 position [27]. By contrast, MEC immortalized by the HPV16E6 mutant Y54D, which is unable to degrade p53, displayed an attenuated p14ARF-induced senescence response and continued to proliferate [28]. Since E6 Y54D retains ability to downregulate the hADA3 protein but not p53, this suggests a role of hADA3 in modulation of p53 activation signals in response to p14ARF. Besides, this highlights a p53-independent role of hADA3 in cellular senescence. Moreover, HPV16E6 L50G mutant, which holds the ability to inhibit p300-mediated p53 acetylation but was unable to degrade hADA3 and p53, failed to achieve immortalization and senescence [27]. These findings underscore the importance of hADA3 in cellular senescence.

### 3.5. Involvement of ADA3 in apoptosis

The contribution of hADA3 in apoptotic pathways remains to be clarified. Several studies have highlighted the significance of hADA3 in p53-mediated DDR, and hence it might play a key role in p53-dependent apoptosis [29,30]. Evidence has shown that depletion of hADA3 caused a defect in perforin/hGrzB-induced Bid cleavage, leading to reduced apoptosis [31]. Additionally, recent identification of hADA3 interaction with AATF further supports its role in apoptosis [8]. AATF is also known to activate p53 functions by means of phosphorylation [8]. Thus, there are indications for hADA3's involvement in apoptosis and detailed investigations are needed to define the mechanisms further.

### 3.6. ADA3, a crucial regulator of DNA damage response and maintenance of genomic stability

Genomic instability being one of the hallmarks of cancer, maintenance of genomic stability is a prerequisite for the flawless propagation of genetic information to daughter cells [32]. This is achieved by highly coordinated events that involve chromatin remodelling, cell cycle checkpoint control, DNA replication, recombination and repair [33]. For instance, loss of hADA3 exhibited chromosomal aberrations such as chromosome breaks, fragments, deletions, translocation and delayed DDR [33]. Loss of ADA3 led to an increase in the levels of DDR proteins, such as pATM, p53BP1, γH2AX and pRAD51. Significantly, Ada3-null cells exhibited a delay in the disappearance of DNA damage foci for several DDR critical proteins after ionizing radiation, suggesting the important role of ADA3 in DDR. Evidence demonstrating increment of basal phosphorylation of various key DDR mediators *viz*. ATM, γH2AX, 53Bp1 and RAD51, as a consequence of disruption of murine Ada3 gene, substantiated its role in cellular genotoxic stress response [33]. Even in the absence of external damaging stress signals, Ada3^−/−^ cells displayed elevated levels of aforementioned DNA damage responsive factors in comparison with Ada3^fl/fl^ cells, suggesting that loss of ADA3 itself might be inducing DNA damage [33]. ADA3-deleted cells also demonstrated persistence of DNA damage [33], fortifying its significance in DNA repair pathways.

DDR is manifested by recruitment of DNA damage repair factors to the site of DNA damage. HATs, in addition to ATP-dependent chromatin remodellers, allow these proteins to access the DNA at the damaged sites. In this context, the role of GCN5 and ADA2 in nucleotide excision repair (NER) was revealed by the finding that deletion of either Ada2 or Gcn5 delays the cyclobutane pyrimidine dimer removal on the MET16 locus, a gene regulated by these two components of the SAGA/ADA complex. Studies have also shown the interaction of STAGA complex with UV-damaged DNA-binding factors DDB1 and DDB2 facilitating the recruitment of NER machinery through the HAT activity of GCN5. Human ADA3 has been identified to interact with p300, PCAF and GCN5 [3,10]. These HATs are known to play a critical role in DNA repair and damage responses such as when GCN5, in coordination with E2F1, recruits excision repair factors following UV-mediated DNA damage [34,35]. The role of p300 in DDR has also been documented, where it has been shown to stabilize and trans-activate p53 in response to DNA damage. Other HATs such as MOF acetylates H4 K16 and mediates recruitment of repair proteins, such as Mdc1, 53BP1 and Brca1, upon ionizing radiation-induced DNA damage.

**Figure 3.**
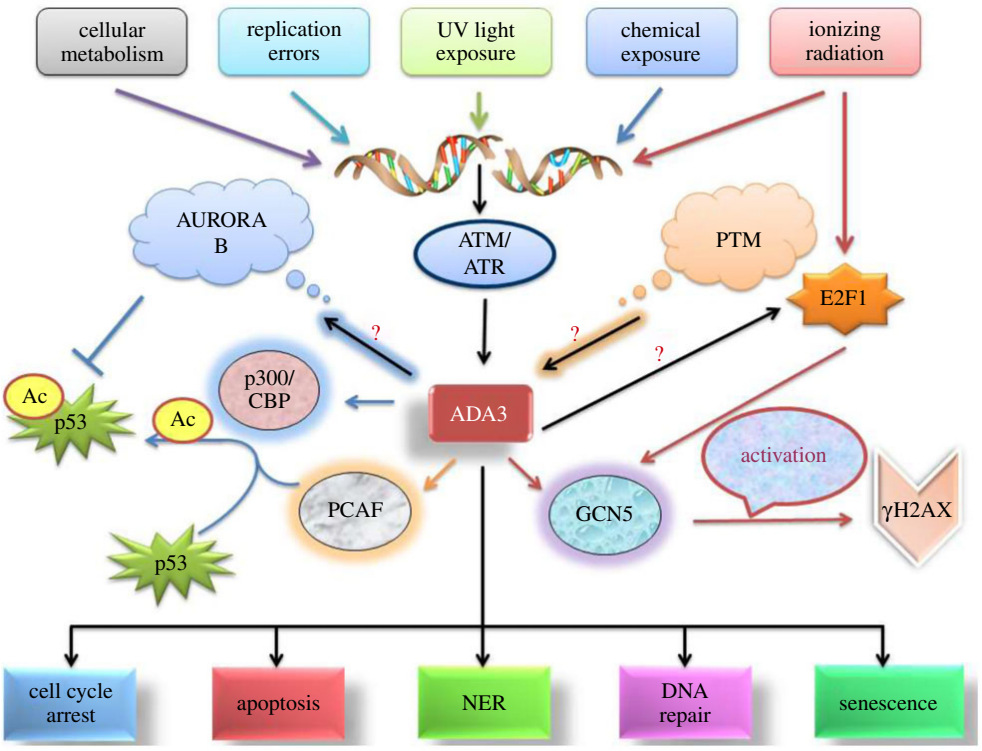
The role of hADA3 in DDR. As a consequence of DNA damage, hADA3 gets activated, followed by initiation of a downstream activation cascade of key mediators of DDR regulated via p300/CBP complex, PCAF and GCN5. The ultimate fate is either DNA repair, if possible, or cellular death.

Additionally, recently identified hADA3-interacting partners PPP2R5D and PPP1R7 are recognized for their roles in the regulation of DNA replication, transcription and cell division [8]. More precisely, PPP1R7 is the regulatory component of PP1, which in turn interacts with AURORA-B, a crucial chromosome stabilizer during mitosis [36,37]. Although the localization of hADA3 of ATAC complex with AURORA-B does not occur at the time of mitosis, the hADA3-dependent interaction and acetylation of α-tubulin play crucial roles in the normal separation of chromosomes and cell division. As the AURORA-B-interacting protein TACC3 is critical for microtubule-dependent centrosome stabilization and has been shown to interact with GCN5 and PCAF complexes containing hADA3, an ADA3-AURORA B-associated mechanism to guard the genomic integrity can be envisioned. This could provide a possible explanation for the genomic instability in hAda3 KO MEFs as well as embryonic lethality [6]. Altogether, these findings point towards the functional participation of HATs in the maintenance of genomic integrity and open up a new avenue in hADA3 research. Nevertheless, extensive investigations are required to decipher the underlying molecular mechanism for participation of hADA3 in cell cycle checkpoint control, DNA repair and maintenance of genomic integrity [38]. DNA damage signalling and sensing involves a complex array of proteins whose action at the DNA damage site is regulated by post-translational modifications (PTM) including phosphorylation, ubiquitination, SUMOylation, neddylation, acetylation and methylation. There have been data to verify PTM of hADA3 in response to DNA damage, suggested by the presence of high molecular weight bands on SDS-PAGE under denaturing conditions. This increment in the size of the protein is due to multiple covalent modifications. DNA damage activation of cell cycle checkpoints is principally regulated by the ATM and ATR kinases. Their function, in turn, is mediated by phosphorylation and activation of cell cycle checkpoint proteins CHK1, CHK2 and p53. Exposure of cells to caffeine, an inhibitor of ATM and ATR kinases, is expected to rescue cells from S-phase and G2/M checkpoint response to DNA damage. The study shows attenuated accumulation of slower migrating forms of hADA3 in cells treated with caffeine prior to DNA damage. This shows a role of ATM and ATR kinase-mediated phosphorylation of hADA3 in response to DNA damage. Overall, this proposes hADA3 is phosphorylated by DNA damage-activated ATM and ATR kinases, and this, in turn, may provide future opportunities for further modifications [39] (figure 3).

### 3.7. Unfolding the role of ADA3 in development

Embryogenesis is defined as the phase of prenatal development accompanied by establishment of the characteristic configuration of the embryonic body. In humans, this phenomenon is deemed as the period from the formation of the embryonic disc in the second week, to the time the conceptus is usually regarded as a fetus around the end of eighth week. Precise development and maintenance of organ homeostasis demands tightly regulated cell cycle entry and progression, and there are many overseers affiliated to these functions, a notable player being ADA3. ADA3 is an established modulator of growth in yeast as well as *Drosophila*, where ADA3 deficiency causes arrest in early development. ADA3 is essential for embryonic development in mice, as concluded from the studies that show Ada3-null embryos undergo early lethality. Developmental block at an early embryonic stage imposed by ADA3 erasure results in arrest of development at the blastocyst stage. Extensive cellular proliferation being a characteristic of the early stage of embryogenesis, a potential role of ADA3 in cell proliferation was insinuated as Ada3^−/−^ embryos failed to remain viable beyond E3.5. This observed embryonic lethality of Ada3^−/−^ mice alluded a latent role of ADA3 in tissue growth and development. ADA3 was found to be ubiquitously expressed in all the tissues with preference in the mammary gland, lung and thymus, which conjured up ubiquitous functional roles of ADA3. Clear evidence of the indispensable role of ADA3 in cell proliferation by promotion of G1 to S as well as G2/M to G1 cell cycle progression came from studies using the conditional deletion feature of the mouse model by using Cre-dependent gene deletion in MEFs from Ada3^fl/fl^ mice. Reconstitution with exogenous hADA3 rescued MEFs from the Ada3^−/−^ phenotype that led to reduced proliferative rate. Preliminary analyses of alterations in the levels of core components of mammalian cell cycle machinery publicized reduction in hypophosphorylated Rb on ADA3 deletion. ADA3 deficiency-mediated G1/S blockage was intensified by p27 knockdown, hinting at the involvement of the latter. p27 level is regulated by cell cycle-associated c-Myc, transcription of which is ADA3-dependent, thus manifesting ADA3 as an inspector of p27 stability. Thus, the early embryonic lethality of Ada3-deficient mice might be due to faulty c-Myc modulation by ADA3-containing complexes. Furthermore, mammalian embryogenesis is characterized by highly dynamic DNA methylation [40], and ADA3 is already an established regulator of epigenetic modification, thus strengthening its contribution to the development of the embryo. Accordingly, probing into the mechanistic role of ADA3 in embryonic development, using cell type and stage-specific conditional depletion of ADA3, will enlighten us about its functional role in physiological and pathological settings.

### 3.8. Regulation of ADA3, a neglected aspect

**Figure 4.**
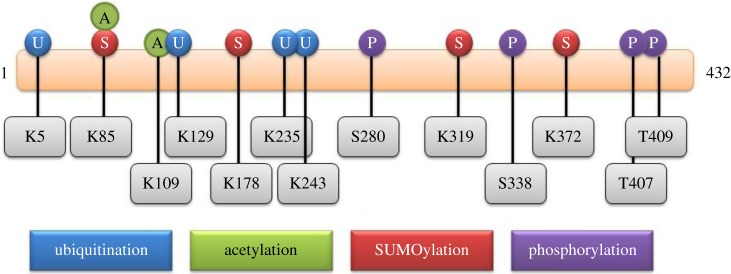
*In silico* prediction of potential sites of hADA3 PTM. Different PTM of hADA3 have been depicted with possible residues of modification for ubiquitination, acetylation, SUMOylation and phosphorylation.

Considering the critical role of this multi-dimensional co-activator protein in various biological processes, it is likely that hADA3 cellular levels must be tightly regulated. However, the mechanisms for regulation of ADA3 are poorly understood. Since post-translational amendments are known to play a central role in imparting dynamic functions to proteins and creating diversity in signalling, the possible modifications of hADA3 were studied *in silico* (figure 4). So far, there has only been a single report proposing phosphorylation of hADA3 upon DNA damage [39]. Bioinformatics analysis of hADA3 primary sequence reveals potential sites for PTM, which were found to be conserved across species.

The tools employed to predict the potential PTM sites are as follows: for phosphorylation, disorder-enhanced phosphorylation sites predictor (DISPHOS) [41], NetPhos v. 2.0 [42] and Phosida [43]; for ubiquitination, UbPred [44], BDM-PUB (http://bdmpub.biocuckoo.org) and CKSAAP [45]; for SUMOylation, SUMOplot (http://www.abgent.com/sumoplot) and SUMOsp v. 2.0 [46]; for acetylation, ASEB [47] and Phosida [43]; for nitrosylation, GPS-SNO v. 1.0 [48] and iSNO-PseAAC [49]. hADA3 functions are regulated by these modifications as a result of alteration in their interaction with other proteins, subcellular localization and stability. Previous reports have exhibited stabilization and appearance of high molecular weight forms of hADA3 after treatment with Adriamycin. However, caffeine treatment led to disappearance of these high-molecular-weight species, implying that hADA3 could undergo phosphorylation under DNA damaging condition [39]. This strongly supports the notion that phosphorylated hADA3 may participate in DNA repair functions. More testimony of the role of hADA3 in DDR comes from its functional association with STAGA and PCAF complexes which have been shown to interact with DNA damage sensory proteins such as DDB1 and DDB2 [9].

Cellular systems with broad and important regulatory functions such as the ubiquitin superfamily are susceptible targets of viruses. This superfamily is characterized by small protein modifiers that are covalently attached to protein substrates through a series of biochemically similar steps. These modifiers include ubiquitin and a series of ubiquitin-like proteins (Ubls), such as SUMOs and ISG15. These proteins have been implicated as modulators of HPV-mediated viral processes. Recent studies on individual HPV early proteins have shown that ubiquitination is a key element in targeting these proteins to the proteasome. Redirecting the ubiquitous PTM systems such as ubiquitination and SUMOylation provide the virus with enormous opportunities for reprogramming the cellular environment to favour viral persistence and reproduction. For HPVs, since none of the viral proteins has intrinsic E3 ligase or deubiquitinating activity, manipulation of these Ubl systems requires interaction between HPV proteins and cellular components of the ubiquitin or SUMO pathways [50]. HPV16 E6 targets hADA3 for proteasomal degradation via E6AP ubiquitin ligase, contributing to low levels of hADA3 in HPV-positive cervical cancer cell lines. hADA3 also undergoes increased SUMOylation in the presence of HPV16 E6. This is the first evidence indicating that hADA3 gets post-translationally modified by SUMOylation that de-stabilizes it and establishes a link between SUMOylation and E6-mediated ubiquitination of hADA3 [51].

Recent identifications of hADA3 interactions with PP2A and PP1 phosphatase may be an indication for their involvement in hADA3 de-phosphorylation. PTM like ubiquitination, SUMOylation and acetylation require lysine residues; thus, these modifications can influence or compete with each other. Additionally, prior modification on one site can also affect the pattern of other subsequent modifications by altering accessibility of other lysine residues on hADA3. As hADA3 is present in several HAT complexes, acetylation of hADA3 remains a strong possibility, which in turn can influence its stability, localization and thus its functions. All this underscores the importance of such PTM-related investigations on hADA3 and is expected to shed light on the molecular mechanisms of diverse cellular functions of hADA3.

### 3.9. ADA3: its association with cancer

Recently Mirza *et al*. [52] have proposed that cytoplasmic localization pattern of hADA3 along with ErbB2+/EGFR+ status may serve as a prognostic marker for ER-positive breast cancer. In this study, by analysing hADA3 distribution in breast cancer tumour samples, they demonstrated that predominant nuclear localization of hADA3 in breast cancer tissues correlates with ER expression and together can serve as helpful markers of prognosis, whereas predominant cytoplasmic hADA3 expression correlates with ErbB2+/EGFR+ expression and together may be used for poor prognosis. Additionally, hADA3 has been found to interact with ER and trans-activate its downstream targets, whereas hAda3 knockdown in ER-positive breast cancer cells led to reduced proliferation as well as small colonies in matrigel growth medium, indicating a role of hADA3-ER connection in maintenance of normal proliferation [10]. This also implies that hADA3-ER may control tumour proliferation in oestrogen-dependent manner and any disruption of this control may lead to oncogenic development.

**Figure 5.**
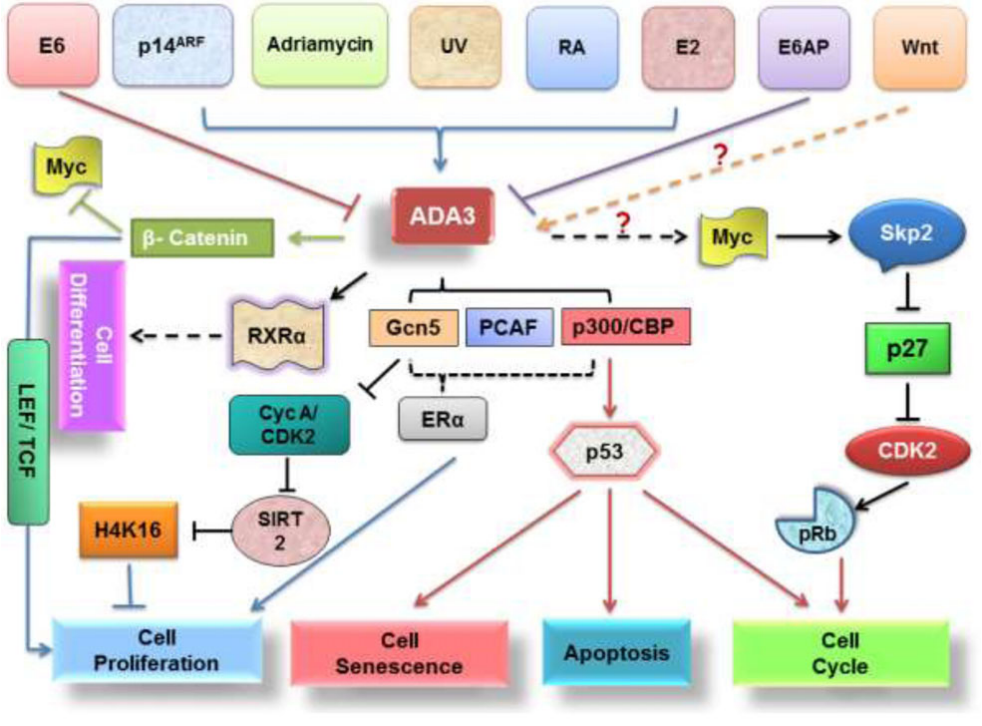
Putative hADA3 signalling pathways. hADA3 functions are regulated through a complex network of signalling pathways, some signals including hormones, genotoxic stress or several classes of proteins like oncoproteins and tumour suppressors, to facilitate an assortment of biological activities related to cell cycle arrest, apoptosis, senescence, cell proliferation and differentiation.

Deregulation of β-catenin, a component of Wnt signalling pathway has been found in multiple cancers. Interestingly, hADA3 has been shown to interact with β-catenin through its Armadillos repeat sequences 6–10 and C-terminal domain thereby enhancing the transcriptional activity of β-catenin response element LEF/TCF *in vitro* [22]. Moreover, reduction in cell proliferation upon hAda3 knockdown suggests its role in the regulation of β-catenin pathway and cancer development [22]. High level of c-Myc (a β-catenin target) in hAda3 null MEFs invokes G2/M-G1 phase arrest by increasing p27 level. Also, acetylation of β-catenin negatively regulates c-Myc, bringing down its proliferation effects, and thus provides a logical explanation for increased proliferation in cells containing hADA3 and β-catenin and cell cycle arrest in Ada3^−/−^ MEFs (figure 5).

**Figure 6.**
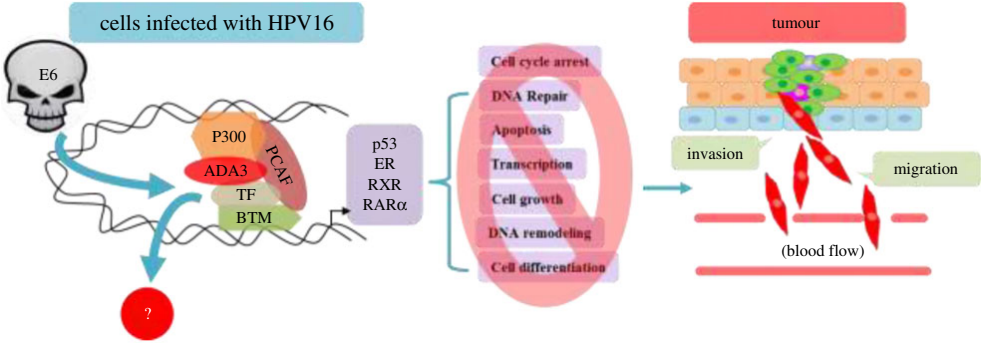
Involvement of HPV16E6 oncoprotein in vitiation of hADA3 mediated functions. HPV16E6 oncoprotein, through its hijacking of hADA3, agitates the normal functions of several transcription modulators such as p300 and PCAF, causing deregulation of numerous TF, thus affecting a multitude of imperative physiological process culminating in tumorigenesis.

In addition to nuclear receptors (ER and RXR), p53 tumour suppressor and β-catenin, ADA3 has also been found to interact with IL-1α [15,17,22,29,53]. IL-1α critically regulates and induces apoptotic machinery, and in yeast, a GAL4/IL-1NTP (IL-1NTP is a proteolytic maturation product of IL-1α) fusion protein was proved to exert a growth inhibitory effect in alliance with an intact SAGA complex [15]. ADA3 has been proved to be a component of SAGA complexes, which, together with prior results, point towards a role of ADA3 in impeding the crucial hallmarks of cancer. Moreover, studies showing inactivation of hADA3 by viral oncoprotein HPV16E6 provide crucial links for its role in carcinogenesis [19]. HATs like p300 and GCN5 are also known to be favourite targets of various viral oncoproteins [54–56], which again suggests a potential role of hADA3 in viral oncogenesis. It is noteworthy that it might be one of the mechanisms for malignancy in cervical cancer where high-risk HPVs interfere with hADA3-mediated signalling and cause uncontrolled cell proliferation (figure 6).

Increasing evidence supports the concept that many viral transforming proteins induce oncogenesis by interacting with and perturbing the function of key cellular proteins involved in the maintenance of normal cellular behaviour. Importantly, many of the viral oncogene targets have been demonstrated to play key roles in human cancer, thus providing a clear rationale for seeking out new targets of viral oncoproteins. Two HPV oncogenes, E6 and E7, have been recognized by *in vitro* studies that are nearly always expressed in HPV-associated carcinomas and cell lines derived from them. The HPV oncoprotein E6 is an essential requirement for oncogenic transformation. HPV oncoprotein E6 alleviates the function of p53 tumour suppressor protein—one way of contributing to oncogenesis. High-risk HPV-E6 has been identified to associate with hADA3 and manipulate the BTM for its own benefit. This implies a crucial contribution of E6-induced hADA3 inactivation towards HPV-mediated oncogenesis. However, the mechanisms involved in degradation of hADA3 by E6 have not yet been investigated. hADA3 had been identified as a novel E6-interacting protein and a target of E6-induced degradation. hADA3 binds to the high-risk (cancer-associated) but not the low-risk HPV E6 proteins and to immortalization-competent but not to immortalization-defective HPV16 E6 mutants, suggesting a role for the perturbation of hADA3 function in E6-mediated oncogenesis [14,57].

p53 is a well-established target of hADA3 co-activator function, which leads to the conclusion that abrogation of hADA3 function provides an additional strategy for HPV E6 to inactivate p53 function [29]. Given the evolutionary conservation of hADA3, it is likely that viral oncoprotein-mediated inactivation of its function plays an important role in oncogenesis. E6–p53 interaction is important for cell cycle progression. p53 is a major transcription factor involved in regulation of several targets associated with cell cycle and apoptosis. E6 binds to p53 and targets it for ubiquitin-mediated degradation. E6-mediated degradation requires another cellular protein, E6-associated protein (E6AP), an E3 ubiquitin ligase. E6AP does not play any role in p53 degradation under normal circumstances; E6AP promotes p53 degradation only in the presence of E6. E6 binding to p53 also blocks p53 translocation to the nucleus and retains it in the cytoplasm—this abrogates p53 function without its degradation and prevents it from activating expression of its target genes. E6 can induce cell cycle arrest in both p53-dependent and -independent manner. E6 binds to Bak, a pro-apoptotic factor, in a p53-independent manner, and degrades it, possibly via E6AP [19,57]. Bak can also bind to E6AP in the absence of E6. Bak is abundantly expressed in the upper layers of the epithelium. Interestingly, HPV is strictly an epitheliotropic virus. E6 can bind to CBP/p300 and mitigate its activation of p53. CBP activates transcription by acetylating histone and non-histone proteins, and by bridging of DNA-bound TF to components of the basal transcription machinery. E6 prevents CBP-mediated acetylation of p53, and therefore reduces its affinity for its cognate DNA-binding sites. E6 may also target the bridging mechanism. This leads to repression of downstream target genes, some of which are responsible for cytokine production and immune signalling. IL-6 and IL-8 promoters are regulated by NF-κB, which in turn is co-activated by CBP/p300. E6 has been shown to inhibit NF-κB transcription. E6 can activate telomerase, a ribonucleoprotein enzyme, essential to maintain the telomeric structures at the end of the chromosomes. Telomerase is active in more than 90% of immortal and cancer cells, but is absent in normal somatic cells. Lack of telomerase activity in normal cells leads to shortening of the telomeric ends and this serves as a ‘mitotic clock’ that is important for regulation of normal lifespan of a cell. Telomerase uses an RNA molecule as a template and a reverse transcriptase protein component for synthesis of DNA. Hence, telomerase activity is closely associated with expression of the catalytic subunit, hTERT. E6 has been reported to induce telomerase activity supposedly via c-Myc-mediated transcriptional activation of hTERT, thereby promoting immortalization of the cells. c-Myc remains elevated in cells expressing E6; c-Myc happens to be a target gene of the Wnt pathway that is closely associated with hADA3 [27].

Cancer, being an insidious ailment, consumes innumerable lives all over the world. The main challenge is to come up with innovative therapeutic approaches with enhanced sensitivity and flawless specificity. Although ADA3 was initially recognized simply as a well-conserved transcriptional co-activator, intensive research over the past few years has shed light on its cellular functions and established ADA3 as a fundamental milestone of cancer initiation and progression. An interesting finding is the myriad of PTM associated with this protein, consistent with the fact that such a multifaceted protein demands tight regulation. It is evident that ADA3 is a critical regulator of a host of diverse biological processes, and that deregulation of ADA3 is a potentially significant cause behind tumorigenesis. Contemporary data suggest the use of ADA3 as a biomarker in breast cancer diagnosis. Its association with β-catenin has also ascertained its role in carcinogenesis. Being a substrate of transforming retroviral oncoproteins, its contribution to malignancy is irrevocable. In addition, ICGC (https://dcc.icgc.org) and cBioportal repository (http://www.cbioportal.org) data have reproducibly put forth an assorted array of hADA3 alterations, including overexpression, point mutations, deletion mutations and frameshift mutations, as contributing factors to many diverse cancers. A perception that demands some attention is our own *in silico* finding of hADA3 ubiquitination site K129, found to be mutated in colorectal carcinoma with a peculiarly high incidence rate (http://www.cbioportal.org). These data, put together, provide credible importance to hADA3 as a pivotal player in various cancers. All recent evidence points out the indispensable role of ADA3 as a promising therapeutic target in cancer treatment. An intriguing avenue to explore would be the regulation of ADA3; although a lot of work is currently in progress focusing on this particular aspect, more rigorous studies are an exigency to clearly understand the mechanisms and role of unidentified factors coupled to this process.

### 3.10. Future directions of hADA3 research

Multiple investigations in varied arenas in the recent past led to recognition of the functional significance of the transcriptional co-activator, hADA3. The journey of hADA3 discovery began with identification of its importance in transcriptional co-activator systems involving various HATs. Insights from genetically manipulated mouse model delineated its central role in cell cycle control. Knockout studies further unfolded its physiological significance in embryonic development, acetylation of core histones, gene expression, etc. Observations from overexpression and depletion studies confirm the role of hADA3 in reducing the proliferative characteristics of cancer cells. Additionally, hADA3 deficiency leading to acetylation defects in p53 after DNA damage is an intriguing observation. Consequently, it can be perceived that hADA3 may act as a tumour suppressor. In the same line, more recently, hADA3 has been reported to have some implications in HPV-related cancer and one report even highlights its prognostic importance. Furthermore, owing to its ability to maintain the sophisticated balance between cell proliferation and differentiation, malfunction of hADA3 can be speculated to cause various human diseases, including cancers, and its level may correlate with disease prognosis. This necessitates further elaborate studies with transgenic and knockout mice in order to delineate the important roles of ADA3. Exploring the significance of PTM, its correlation with hADA3 subcellular localization and functions remain other interesting aspects to work upon. Equally important would be to understand its regulatory pathways, the factors responsible for perturbing the endogenous levels of hADA3 and, therefore, its correspondence to diseases. The increasing association of ADA3 with various cancers and its role in embryogenesis, as well as poor prognosis for breast tumours, tempts us to speculate its prospective role in cellular stemness and regenerative processes. Clearly, much remains to be learned about hADA3 and undertaking more intricate studies can warrant decoding of numerous elusive disease mechanisms. Overall, the impact of hADA3 on health and disease provides a potential perspective for future research. Apart from advancing the basic understanding of hADA3 biology such investigations will reveal the therapeutic potential of hADA3 in future clinical treatments.
